# (2*E*)-2-[(3-Methyl-5-phen­oxy-1-phenyl-1*H*-pyrazol-4-yl)methyl­idene]hydrazinecarbothio­amide

**DOI:** 10.1107/S1600536812026931

**Published:** 2012-06-20

**Authors:** Hoong-Kun Fun, Ching Kheng Quah, Shobhitha Shetty, Balakrishna Kalluraya, Nitinchandra

**Affiliations:** a X-ray Crystallography Unit, School of Physics, Universiti Sains Malaysia, 11800 USM, Penang, Malaysia; b Department of Studies in Chemistry, Mangalore University, Mangalagangotri, Mangalore 574 199, India

## Abstract

In the title compound, C_18_H_17_N_5_OS, the mean plane of the pyrazole ring [maximum deviation = 0.0031 (12) Å] forms dihedral angles of 19.6 (4) and 9.3 (5)° with the two disorder components of the N-bound benzene ring (with equal occupancies for the two orientations) and a dihedral angle of 72.58 (8)° with the C—O-bonded benzene ring. The mol­ecule exists in a *trans* conformation with respect to the N=C bond [1.2792 (19) Å]. The mol­ecular structure features an intra­molecular C—H⋯O hydrogen bond, forming an *S*(6) ring. In the crystal, N—H⋯N and N—H⋯S hydrogen bonds result in the formation of zigzag layers lying parallel to (10-1).

## Related literature

For general background to and applications of the pyrazole derivatives, see: Rai *et al.* (2008 ▶); Isloor *et al.* (2009 ▶); Girisha *et al.* (2010 ▶). For standard bond-length data, see: Allen *et al.* (1987 ▶). For the stability of the temperature controller used in the data collection, see Cosier & Glazer (1986 ▶). For hydrogen-bond motifs, see: Bernstein *et al.* (1995 ▶). For related structures, see: Fun *et al.* (2011*a* ▶,*b* ▶,*c* ▶).
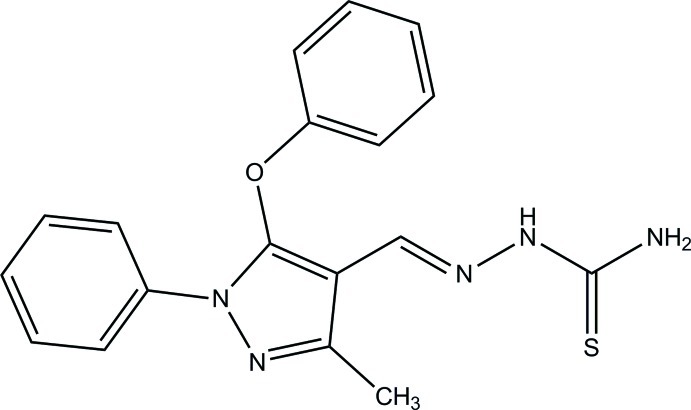


## Experimental

### 

#### Crystal data

C_18_H_17_N_5_OS
*M* *_r_* = 351.43
Monoclinic, 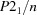

*a* = 8.8280 (1) Å
*b* = 10.8519 (2) Å
*c* = 17.7353 (2) Å
β = 94.379 (1)°
*V* = 1694.09 (4) Å^3^
*Z* = 4
Mo *K*α radiation
μ = 0.21 mm^−1^
*T* = 100 K
0.29 × 0.27 × 0.22 mm

#### Data collection

Bruker SMART APEXII CCD diffractometer
Absorption correction: multi-scan (*SADABS*; Bruker, 2009 ▶) *T*_min_ = 0.942, *T*_max_ = 0.955
18467 measured reflections
4948 independent reflections
3879 reflections with *I* > 2σ(*I*)
*R*_int_ = 0.042

#### Refinement

*R*[*F* ^2^ > 2σ(*F* ^2^)] = 0.046
*wR*(*F* ^2^) = 0.113
*S* = 1.06
4948 reflections
294 parameters
216 restraints
H atoms treated by a mixture of independent and constrained refinement
Δρ_max_ = 0.43 e Å^−3^
Δρ_min_ = −0.26 e Å^−3^

### 

Data collection: *APEX2* (Bruker, 2009 ▶); cell refinement: *SAINT* (Bruker, 2009 ▶); data reduction: *SAINT*; program(s) used to solve structure: *SHELXTL* (Sheldrick, 2008 ▶); program(s) used to refine structure: *SHELXTL*; molecular graphics: *SHELXTL*; software used to prepare material for publication: *SHELXTL* and *PLATON* (Spek, 2009 ▶).

## Supplementary Material

Crystal structure: contains datablock(s) global, I. DOI: 10.1107/S1600536812026931/hb6849sup1.cif

Structure factors: contains datablock(s) I. DOI: 10.1107/S1600536812026931/hb6849Isup2.hkl

Supplementary material file. DOI: 10.1107/S1600536812026931/hb6849Isup3.cml

Additional supplementary materials:  crystallographic information; 3D view; checkCIF report

## Figures and Tables

**Table 1:** Hydrogen-bond geometry (Å, °)

*D*—H⋯*A*	*D*—H	H⋯*A*	*D*⋯*A*	*D*—H⋯*A*
N1—H1*N*1⋯N5^i^	0.87 (2)	2.50 (2)	3.3237 (19)	158.1 (18)
N2—H1*N*2⋯S1^ii^	0.89 (2)	2.56 (2)	3.4414 (13)	167.7 (17)
C13—H13*A*⋯O1	0.95	2.22	2.814 (12)	120
C13—H13*B*⋯O1	0.79	2.28	2.814 (12)	125

Symmetry codes: (i) 
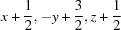
; (ii) 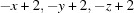.

**Fig. 1.**
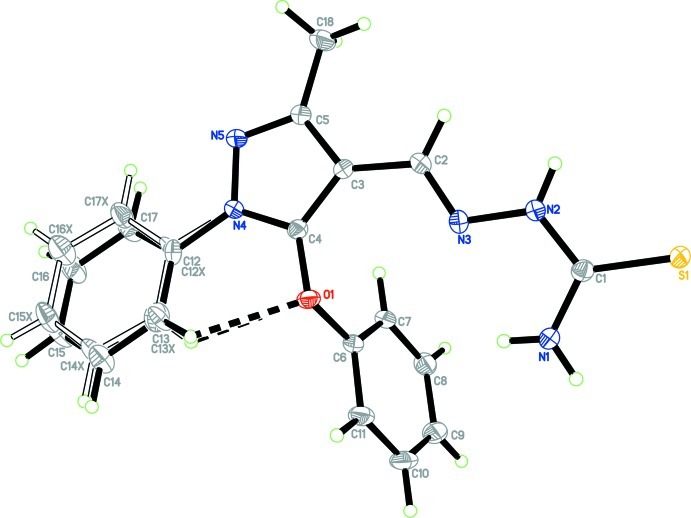
The molecular structure of the title compound showing 50% probability displacement ellipsoids for non-H atoms. Intramolecular hydrogen bonds are shown as dashed lines.

**Fig. 2.**
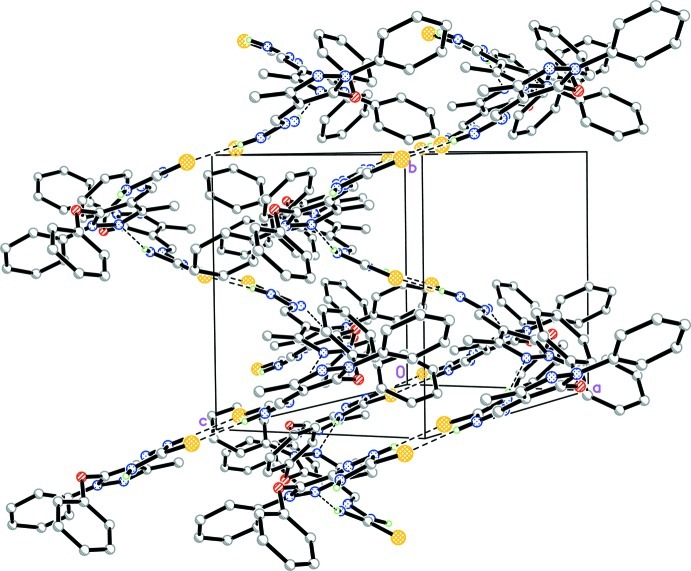
The crystal structure of the title compound, viewed along the [101] direction. H atoms not involved in hydrogen bonds (dashed lines) have been omitted for clarity. Only one disordered component is shown.

## supplementary crystallographic information

### Comment

Pyrazoles possess a wide variety of applications in the agrochemical and pharmaceutical industries including antibacterial (Rai *et al.*, 2008), anti-inflammatory and analgesic (Isloor *et al.*, 2009) activities. In view of these observations and in continuation of our search for biologically active pyrazole derivatives, we herein report the crystal structure of 3-methyl-5-phenoxy-1-phenyl-1H-pyrazole-4-carbaldehyde. Reaction of 5-chloro-3-methyl-1-phenyl-1H-pyrazole-4-carbaldehyde with phenol afforded 5-chloro-3-methyl-1- phenyl-1H-pyrazole-4-carbaldehyde (Girisha *et al.*, 2010).

In the title molecule, Fig. 1, the mean plane of pyrazole ring (N4/N5/C3-C5, maximum deviation = 0.0031 (12) Å at atom N4) forms dihedral angles of 19.6 (4), 9.3 (5) and 72.58 (8)° with the three benzene rings (C12-C17, C12X-C17X and C6-C11). One of the benzene rings (C12-C17) is disordered over two positions with equal refined site-occupancies [0.50 (2) and 0.50 (2)]. The title molecule exists in a *trans* conformation with respect to the N3//dbC2 bond [1.2792 (19) Å]. Bond lengths (Allen *et al.*, 1987) and angles are within normal ranges and are comparable to related structures (Fun *et al.*, 2011*a*, 2011*b*, 2011*c*). The molecular structure is stabilized by intramolecular C13–H13A···O1 and C13–H13B···O1 hydrogen bonds (Table 1), which generate *S*(6) ring motifs (Fig. 2, Bernstein *et al.*, 1995).

In the crystal (Fig.2), intermolecular N1–H1N1···N5 and N2–H1N2···S1 hydrogen bonds (Table 1) result in the formation of zigzag layers parallel to (101).

### Experimental

The title compound was obtained by refluxing a mixture 3-methyl-5-phenoxy-1-phenyl-1*H*-pyrazole-4-carbaldehyde (0.01 mol), thiosemicarbazide (0.01 mol) in ethanol (30 ml) and 3 drops of concentrated sulfuric acid for 1 h. Excess ethanol was removed from the reaction mixture under reduced pressure. The solid product obtained was filtered, washed with ethanol and dried. Pink blocks were obtained by the slow evaporation of an ethanol-*N*,*N*-dimethylformamide (DMF) (3:1) solution.

### Refinement

The N-bound hydrogen atoms were located in a difference Fourier map and refined freely [N–H = 0.81 (2)-0.89 (2) Å]. The rest of hydrogen atoms were positioned geometrically and refined using a riding model with C–H = 0.95 or 0.98 Å and *U*_iso_(H) = 1.2 or 1.5 *U*_eq_(C). A rotating-group model was applied for the methyl group. One of the benzene rings (C12-C17) is disordered over two positions with equal refined site-occupancies [0.50 (2) and 0.50 (2)]. Similarity and rigid-bond restraints were applied to the disordered atoms.

### Crystal data

**Table:**

C_18_H_17_N_5_OS	*F*(000) = 736
*M**_r_* = 351.43	*D*_x_ = 1.378 Mg m^−^^3^
Monoclinic, *P*2_1_/*n*	Mo *K*α radiation, λ = 0.71073 Å
Hall symbol: -P 2yn	Cell parameters from 5058 reflections
*a* = 8.8280 (1) Å	θ = 2.3–32.4°
*b* = 10.8519 (2) Å	µ = 0.21 mm^−^^1^
*c* = 17.7353 (2) Å	*T* = 100 K
β = 94.379 (1)°	Block, pink
*V* = 1694.09 (4) Å^3^	0.29 × 0.27 × 0.22 mm
*Z* = 4	

### Data collection

**Table:**

Bruker SMART APEXII CCD diffractometer	4948 independent reflections
Radiation source: fine-focus sealed tube	3879 reflections with *I* > 2σ(*I*)
Graphite monochromator	*R*_int_ = 0.042
φ and ω scans	θ_max_ = 30.0°, θ_min_ = 2.2°
Absorption correction: multi-scan (*SADABS*; Bruker, 2009)	*h* = −12→12
*T*_min_ = 0.942, *T*_max_ = 0.955	*k* = −15→15
18467 measured reflections	*l* = −22→24

### Refinement

**Table:**

Refinement on *F*^2^	Primary atom site location: structure-invariant direct methods
Least-squares matrix: full	Secondary atom site location: difference Fourier map
*R*[*F*^2^ > 2σ(*F*^2^)] = 0.046	Hydrogen site location: inferred from neighbouring sites
*wR*(*F*^2^) = 0.113	H atoms treated by a mixture of independent and constrained refinement
*S* = 1.06	*w* = 1/[σ^2^(*F*_o_^2^) + (0.0482*P*)^2^ + 0.7836*P*] where *P* = (*F*_o_^2^ + 2*F*_c_^2^)/3
4948 reflections	(Δ/σ)_max_ = 0.001
294 parameters	Δρ_max_ = 0.43 e Å^−^^3^
216 restraints	Δρ_min_ = −0.26 e Å^−^^3^

### Special details

**Table:**

Experimental. The crystal was placed in the cold stream of an Oxford Cryosystems Cobra open-flow nitrogen cryostat (Cosier & Glazer, 1986) operating at 100.0 (1) K.
Geometry. All esds (except the esd in the dihedral angle between two l.s. planes) are estimated using the full covariance matrix. The cell esds are taken into account individually in the estimation of esds in distances, angles and torsion angles; correlations between esds in cell parameters are only used when they are defined by crystal symmetry. An approximate (isotropic) treatment of cell esds is used for estimating esds involving l.s. planes.
Refinement. Refinement of F^2^ against ALL reflections. The weighted R-factor wR and goodness of fit S are based on F^2^, conventional R-factors R are based on F, with F set to zero for negative F^2^. The threshold expression of F^2^ > 2sigma(F^2^) is used only for calculating R-factors(gt) etc. and is not relevant to the choice of reflections for refinement. R-factors based on F^2^ are statistically about twice as large as those based on F, and R- factors based on ALL data will be even larger.

### Fractional atomic coordinates and isotropic or equivalent isotropic displacement parameters (Å^2^)

**Table:**

	*x*	*y*	*z*	*U*_iso_*/*U*_eq_	Occ. (<1)
S1	1.02274 (4)	0.97510 (4)	1.12626 (2)	0.01906 (10)	
O1	0.32416 (12)	0.80260 (10)	1.01785 (6)	0.0171 (2)	
N1	0.75067 (17)	0.87882 (14)	1.14880 (8)	0.0208 (3)	
N2	0.78833 (15)	0.93939 (12)	1.02818 (7)	0.0168 (3)	
N3	0.64788 (14)	0.89082 (12)	1.00657 (7)	0.0169 (3)	
N4	0.24554 (14)	0.73854 (11)	0.89336 (7)	0.0153 (2)	
N5	0.29680 (15)	0.73976 (12)	0.82182 (7)	0.0182 (3)	
C1	0.84379 (17)	0.92800 (13)	1.10068 (8)	0.0160 (3)	
C2	0.61467 (17)	0.88237 (14)	0.93528 (9)	0.0179 (3)	
H2A	0.6840	0.9127	0.9013	0.022*	
C3	0.47312 (17)	0.82734 (14)	0.90555 (8)	0.0163 (3)	
C4	0.35025 (17)	0.78988 (13)	0.94386 (8)	0.0151 (3)	
C5	0.43228 (18)	0.79306 (14)	0.82963 (8)	0.0183 (3)	
C6	0.40260 (17)	0.72594 (14)	1.07080 (8)	0.0155 (3)	
C7	0.50846 (17)	0.63938 (14)	1.05158 (8)	0.0167 (3)	
H7A	0.5306	0.6288	1.0004	0.020*	
C8	0.58163 (19)	0.56845 (15)	1.10841 (9)	0.0207 (3)	
H8A	0.6548	0.5090	1.0961	0.025*	
C9	0.5488 (2)	0.58360 (16)	1.18318 (9)	0.0250 (4)	
H9A	0.5994	0.5350	1.2219	0.030*	
C10	0.4411 (2)	0.67053 (17)	1.20104 (9)	0.0269 (4)	
H10A	0.4180	0.6807	1.2521	0.032*	
C11	0.36739 (19)	0.74238 (16)	1.14485 (9)	0.0219 (3)	
H11A	0.2941	0.8018	1.1570	0.026*	
C12	0.1022 (18)	0.687 (2)	0.9077 (9)	0.0163 (15)	0.50 (2)
C13	0.0307 (13)	0.7157 (11)	0.9729 (7)	0.0168 (12)	0.50 (2)
H13A	0.0743	0.7740	1.0082	0.020*	0.50 (2)
C14	−0.1050 (15)	0.6578 (12)	0.9854 (7)	0.0217 (13)	0.50 (2)
H14A	−0.1526	0.6748	1.0305	0.026*	0.50 (2)
C15	−0.1725 (12)	0.5757 (10)	0.9335 (7)	0.0263 (15)	0.50 (2)
H15A	−0.2656	0.5368	0.9431	0.032*	0.50 (2)
C16	−0.1035 (11)	0.5501 (10)	0.8673 (6)	0.0301 (15)	0.50 (2)
H16A	−0.1515	0.4966	0.8304	0.036*	0.50 (2)
C17	0.0365 (12)	0.6033 (10)	0.8553 (6)	0.0248 (15)	0.50 (2)
H17A	0.0869	0.5825	0.8116	0.030*	0.50 (2)
C12X	0.0983 (19)	0.683 (2)	0.9013 (9)	0.0186 (16)	0.50 (2)
C13X	0.0278 (15)	0.6967 (13)	0.9686 (8)	0.0259 (17)	0.50 (2)
H13B	0.0763	0.7409	1.0099	0.031*	0.50 (2)
C14X	−0.1156 (15)	0.6440 (14)	0.9742 (8)	0.0276 (16)	0.50 (2)
H14B	−0.1636	0.6502	1.0202	0.033*	0.50 (2)
C15X	−0.1871 (12)	0.5835 (11)	0.9137 (7)	0.0286 (15)	0.50 (2)
H15B	−0.2846	0.5481	0.9177	0.034*	0.50 (2)
C16X	−0.1171 (11)	0.5744 (9)	0.8473 (7)	0.0304 (14)	0.50 (2)
H16B	−0.1681	0.5338	0.8052	0.036*	0.50 (2)
C17X	0.0263 (12)	0.6231 (11)	0.8405 (6)	0.0244 (15)	0.50 (2)
H17B	0.0741	0.6150	0.7946	0.029*	0.50 (2)
C18	0.5243 (2)	0.80836 (19)	0.76296 (10)	0.0300 (4)	
H18A	0.4574	0.8316	0.7186	0.045*	
H18B	0.5754	0.7305	0.7529	0.045*	
H18C	0.6004	0.8730	0.7736	0.045*	
H2N1	0.669 (2)	0.8510 (18)	1.1330 (11)	0.025 (5)*	
H1N1	0.781 (2)	0.8649 (19)	1.1957 (12)	0.030 (5)*	
H1N2	0.850 (2)	0.9640 (19)	0.9934 (12)	0.032 (6)*	

### Atomic displacement parameters (Å^2^)

**Table:**

	*U*^11^	*U*^22^	*U*^33^	*U*^12^	*U*^13^	*U*^23^
S1	0.01562 (19)	0.0281 (2)	0.01348 (17)	−0.00380 (15)	0.00110 (13)	−0.00271 (14)
O1	0.0174 (5)	0.0224 (5)	0.0119 (5)	0.0045 (4)	0.0031 (4)	0.0019 (4)
N1	0.0161 (7)	0.0320 (8)	0.0139 (6)	−0.0047 (6)	−0.0007 (5)	0.0019 (5)
N2	0.0146 (6)	0.0214 (6)	0.0144 (6)	−0.0043 (5)	0.0004 (5)	0.0009 (5)
N3	0.0129 (6)	0.0199 (6)	0.0177 (6)	−0.0019 (5)	−0.0005 (5)	0.0006 (5)
N4	0.0130 (6)	0.0199 (6)	0.0133 (6)	−0.0011 (5)	0.0022 (5)	0.0012 (5)
N5	0.0162 (6)	0.0260 (7)	0.0125 (6)	−0.0030 (5)	0.0020 (5)	0.0012 (5)
C1	0.0166 (7)	0.0167 (7)	0.0147 (7)	0.0011 (6)	0.0020 (5)	−0.0023 (5)
C2	0.0147 (7)	0.0230 (7)	0.0164 (7)	−0.0027 (6)	0.0033 (6)	0.0012 (6)
C3	0.0150 (7)	0.0209 (7)	0.0129 (6)	−0.0018 (6)	−0.0002 (5)	0.0023 (5)
C4	0.0143 (7)	0.0176 (7)	0.0136 (6)	0.0018 (5)	0.0012 (5)	0.0020 (5)
C5	0.0164 (7)	0.0234 (7)	0.0149 (7)	−0.0034 (6)	0.0008 (6)	0.0021 (6)
C6	0.0149 (7)	0.0183 (7)	0.0135 (6)	−0.0019 (5)	0.0020 (5)	0.0035 (5)
C7	0.0165 (7)	0.0194 (7)	0.0148 (7)	−0.0020 (6)	0.0039 (6)	0.0013 (5)
C8	0.0187 (8)	0.0206 (7)	0.0229 (8)	0.0009 (6)	0.0024 (6)	0.0039 (6)
C9	0.0234 (8)	0.0315 (9)	0.0200 (8)	0.0017 (7)	0.0005 (6)	0.0102 (7)
C10	0.0271 (9)	0.0397 (10)	0.0146 (7)	0.0023 (7)	0.0054 (6)	0.0069 (7)
C11	0.0204 (8)	0.0300 (8)	0.0160 (7)	0.0036 (7)	0.0062 (6)	0.0018 (6)
C12	0.011 (2)	0.016 (2)	0.021 (3)	−0.001 (2)	−0.003 (2)	−0.002 (2)
C13	0.013 (2)	0.013 (3)	0.026 (2)	−0.0041 (17)	0.0103 (18)	−0.0068 (17)
C14	0.020 (2)	0.016 (2)	0.030 (3)	−0.0049 (17)	0.006 (2)	−0.0092 (19)
C15	0.020 (3)	0.023 (2)	0.038 (3)	−0.0064 (19)	0.015 (3)	−0.009 (3)
C16	0.023 (2)	0.031 (3)	0.038 (3)	−0.014 (2)	0.007 (2)	−0.012 (2)
C17	0.020 (2)	0.029 (3)	0.026 (3)	−0.004 (2)	0.010 (2)	−0.007 (2)
C12X	0.011 (3)	0.021 (3)	0.025 (3)	−0.001 (2)	0.008 (3)	0.003 (2)
C13X	0.025 (2)	0.024 (4)	0.028 (3)	−0.004 (2)	−0.001 (2)	−0.003 (2)
C14X	0.018 (3)	0.031 (4)	0.035 (3)	−0.005 (2)	0.013 (3)	−0.003 (3)
C15X	0.016 (2)	0.031 (2)	0.040 (4)	−0.0073 (18)	0.007 (3)	−0.006 (3)
C16X	0.024 (2)	0.031 (3)	0.036 (3)	−0.006 (2)	0.009 (2)	−0.011 (2)
C17X	0.017 (2)	0.030 (3)	0.026 (3)	−0.007 (2)	0.004 (2)	−0.005 (2)
C18	0.0265 (9)	0.0471 (11)	0.0170 (8)	−0.0129 (8)	0.0061 (7)	0.0001 (7)

### Geometric parameters (Å, º)

**Table:**

S1—C1	1.6892 (16)	C10—C11	1.388 (2)
O1—C4	1.3566 (17)	C10—H10A	0.9500
O1—C6	1.3979 (17)	C11—H11A	0.9500
N1—C1	1.340 (2)	C12—C13	1.395 (9)
N1—H2N1	0.81 (2)	C12—C17	1.395 (10)
N1—H1N1	0.87 (2)	C13—C14	1.385 (9)
N2—C1	1.3461 (19)	C13—H13A	0.9500
N2—N3	1.3746 (18)	C14—C15	1.382 (8)
N2—H1N2	0.89 (2)	C14—H14A	0.9500
N3—C2	1.2792 (19)	C15—C16	1.393 (8)
N4—C4	1.3567 (19)	C15—H15A	0.9500
N4—N5	1.3793 (17)	C16—C17	1.395 (8)
N4—C12	1.423 (13)	C16—H16A	0.9500
N4—C12X	1.447 (12)	C17—H17A	0.9500
N5—C5	1.326 (2)	C12X—C17X	1.375 (10)
C2—C3	1.448 (2)	C12X—C13X	1.395 (10)
C2—H2A	0.9500	C13X—C14X	1.400 (9)
C3—C4	1.384 (2)	C13X—H13B	0.9500
C3—C5	1.417 (2)	C14X—C15X	1.370 (8)
C5—C18	1.494 (2)	C14X—H14B	0.9500
C6—C11	1.384 (2)	C15X—C16X	1.375 (8)
C6—C7	1.386 (2)	C15X—H15B	0.9500
C7—C8	1.388 (2)	C16X—C17X	1.386 (9)
C7—H7A	0.9500	C16X—H16B	0.9500
C8—C9	1.389 (2)	C17X—H17B	0.9500
C8—H8A	0.9500	C18—H18A	0.9800
C9—C10	1.393 (2)	C18—H18B	0.9800
C9—H9A	0.9500	C18—H18C	0.9800
			
C4—O1—C6	118.51 (11)	C6—C11—C10	118.82 (15)
C1—N1—H2N1	119.9 (14)	C6—C11—H11A	120.6
C1—N1—H1N1	121.4 (14)	C10—C11—H11A	120.6
H2N1—N1—H1N1	118 (2)	C13—C12—C17	120.4 (9)
C1—N2—N3	119.05 (13)	C13—C12—N4	121.8 (8)
C1—N2—H1N2	119.5 (14)	C17—C12—N4	117.8 (9)
N3—N2—H1N2	120.2 (14)	C14—C13—C12	118.9 (8)
C2—N3—N2	115.90 (13)	C14—C13—H13A	120.6
C4—N4—N5	110.39 (12)	C12—C13—H13A	120.6
C4—N4—C12	127.8 (6)	C15—C14—C13	121.4 (8)
N5—N4—C12	121.8 (5)	C15—C14—H14A	119.3
C4—N4—C12X	132.6 (6)	C13—C14—H14A	119.3
N5—N4—C12X	117.0 (6)	C14—C15—C16	119.7 (8)
C12—N4—C12X	4.8 (10)	C14—C15—H15A	120.1
C5—N5—N4	105.35 (12)	C16—C15—H15A	120.1
N1—C1—N2	116.66 (14)	C15—C16—C17	119.7 (7)
N1—C1—S1	123.78 (12)	C15—C16—H16A	120.1
N2—C1—S1	119.56 (12)	C17—C16—H16A	120.1
N3—C2—C3	121.06 (14)	C16—C17—C12	119.8 (8)
N3—C2—H2A	119.5	C16—C17—H17A	120.1
C3—C2—H2A	119.5	C12—C17—H17A	120.1
C4—C3—C5	103.72 (13)	C17X—C12X—C13X	120.6 (10)
C4—C3—C2	129.01 (14)	C17X—C12X—N4	119.1 (9)
C5—C3—C2	127.19 (14)	C13X—C12X—N4	120.3 (9)
O1—C4—N4	121.53 (13)	C12X—C13X—C14X	119.0 (9)
O1—C4—C3	129.96 (14)	C12X—C13X—H13B	120.5
N4—C4—C3	108.46 (13)	C14X—C13X—H13B	120.5
N5—C5—C3	112.08 (14)	C15X—C14X—C13X	120.3 (9)
N5—C5—C18	120.41 (14)	C15X—C14X—H14B	119.8
C3—C5—C18	127.50 (14)	C13X—C14X—H14B	119.8
C11—C6—C7	121.69 (14)	C14X—C15X—C16X	119.7 (8)
C11—C6—O1	115.15 (13)	C14X—C15X—H15B	120.1
C7—C6—O1	123.15 (13)	C16X—C15X—H15B	120.1
C6—C7—C8	118.85 (14)	C15X—C16X—C17X	121.3 (7)
C6—C7—H7A	120.6	C15X—C16X—H16B	119.4
C8—C7—H7A	120.6	C17X—C16X—H16B	119.4
C7—C8—C9	120.55 (15)	C12X—C17X—C16X	119.0 (8)
C7—C8—H8A	119.7	C12X—C17X—H17B	120.5
C9—C8—H8A	119.7	C16X—C17X—H17B	120.5
C8—C9—C10	119.56 (15)	C5—C18—H18A	109.5
C8—C9—H9A	120.2	C5—C18—H18B	109.5
C10—C9—H9A	120.2	H18A—C18—H18B	109.5
C11—C10—C9	120.53 (15)	C5—C18—H18C	109.5
C11—C10—H10A	119.7	H18A—C18—H18C	109.5
C9—C10—H10A	119.7	H18B—C18—H18C	109.5
			
C1—N2—N3—C2	−166.48 (14)	C8—C9—C10—C11	−0.4 (3)
C4—N4—N5—C5	−0.51 (16)	C7—C6—C11—C10	0.4 (2)
C12—N4—N5—C5	−179.7 (12)	O1—C6—C11—C10	−179.52 (15)
C12X—N4—N5—C5	−179.4 (12)	C9—C10—C11—C6	0.1 (3)
N3—N2—C1—N1	−5.8 (2)	C4—N4—C12—C13	20 (3)
N3—N2—C1—S1	174.23 (10)	N5—N4—C12—C13	−161.4 (14)
N2—N3—C2—C3	177.04 (13)	C12X—N4—C12—C13	−164 (27)
N3—C2—C3—C4	7.7 (3)	C4—N4—C12—C17	−158.3 (11)
N3—C2—C3—C5	−168.53 (16)	N5—N4—C12—C17	21 (2)
C6—O1—C4—N4	107.65 (16)	C12X—N4—C12—C17	18 (23)
C6—O1—C4—C3	−75.5 (2)	C17—C12—C13—C14	1 (3)
N5—N4—C4—O1	178.07 (12)	N4—C12—C13—C14	−176.7 (16)
C12—N4—C4—O1	−2.8 (13)	C12—C13—C14—C15	−2 (2)
C12X—N4—C4—O1	−3.3 (14)	C13—C14—C15—C16	0.0 (18)
N5—N4—C4—C3	0.62 (17)	C14—C15—C16—C17	2.8 (13)
C12—N4—C4—C3	179.7 (13)	C15—C16—C17—C12	−3.7 (16)
C12X—N4—C4—C3	179.3 (14)	C13—C12—C17—C16	2 (3)
C5—C3—C4—O1	−177.63 (15)	N4—C12—C17—C16	179.6 (13)
C2—C3—C4—O1	5.5 (3)	C4—N4—C12X—C17X	−171.0 (11)
C5—C3—C4—N4	−0.45 (16)	N5—N4—C12X—C17X	8 (2)
C2—C3—C4—N4	−177.34 (15)	C12—N4—C12X—C17X	−175 (27)
N4—N5—C5—C3	0.21 (17)	C4—N4—C12X—C13X	12 (3)
N4—N5—C5—C18	178.89 (15)	N5—N4—C12X—C13X	−169.3 (15)
C4—C3—C5—N5	0.15 (18)	C12—N4—C12X—C13X	8 (23)
C2—C3—C5—N5	177.11 (15)	C17X—C12X—C13X—C14X	2 (3)
C4—C3—C5—C18	−178.41 (16)	N4—C12X—C13X—C14X	179.0 (16)
C2—C3—C5—C18	−1.5 (3)	C12X—C13X—C14X—C15X	−2 (2)
C4—O1—C6—C11	−178.56 (14)	C13X—C14X—C15X—C16X	0.2 (19)
C4—O1—C6—C7	1.5 (2)	C14X—C15X—C16X—C17X	1.3 (14)
C11—C6—C7—C8	−0.6 (2)	C13X—C12X—C17X—C16X	−1 (3)
O1—C6—C7—C8	179.31 (14)	N4—C12X—C17X—C16X	−177.6 (14)
C6—C7—C8—C9	0.3 (2)	C15X—C16X—C17X—C12X	−1.0 (17)
C7—C8—C9—C10	0.2 (3)		

### Hydrogen-bond geometry (Å, º)

**Table:**

*D*—H···*A*	*D*—H	H···*A*	*D*···*A*	*D*—H···*A*
N1—H1*N*1···N5^i^	0.87 (2)	2.50 (2)	3.3237 (19)	158.1 (18)
N2—H1*N*2···S1^ii^	0.89 (2)	2.56 (2)	3.4414 (13)	167.7 (17)
C13—H13*A*···O1	0.95	2.22	2.814 (12)	120
C13—H13*B*···O1	0.79	2.28	2.814 (12)	125

Symmetry codes: (i) *x*+1/2, −*y*+3/2, *z*+1/2; (ii) −*x*+2, −*y*+2, −*z*+2.
